# The association of diabetes mellitus and routinely collected patient‐reported outcomes in patients with cancer. A real‐world cohort study

**DOI:** 10.1002/cam4.70246

**Published:** 2024-10-24

**Authors:** Dominik J. Ose, Emmanuel Adediran, Bayarmaa Mark, Krista Ocier, William A. Dunson JR, Cindy Turner, Belinda Taylor, Kim Svoboda, Andrew R. Post, Jennifer Leiser, Howard Colman, Cornelia M. Ulrich, Mia Hashibe

**Affiliations:** ^1^ Department of Family and Preventive Medicine, School of Medicine University of Utah Salt Lake City Utah USA; ^2^ Department of Health and Healthcare Sciences Westsächsische Hochschule Zwickau Zwickau Germany; ^3^ Huntsman Cancer Institute, University of Utah Salt Lake City Utah USA; ^4^ Huntsman Cancer Registry University of Utah Hospital Salt Lake City Utah USA; ^5^ Department of Biomedical Informatics University of Utah Salt Lake City Utah USA; ^6^ Department of Neurosurgery University of Utah Salt Lake City Utah USA; ^7^ Department of Population Health Sciences University of Utah Salt Lake City Utah USA

**Keywords:** clinical management, epidemiology and prevention, medical oncology, quality of life

## Abstract

**Objective:**

Current studies have indicated that diabetes mellitus (DM) is highly prevalent in patients with cancer, but there is little research on consequences on the well‐being of patients during cancer treatment. This analysis evaluates the relationship between DM and patient‐reported outcomes (PRO) in patients with cancer, using a large and well‐characterized cohort.

**Methods:**

This study utilized the Total Cancer Care protocol at the University of Utah Huntsman Cancer Institute. For this analysis, we integrated data from electronic health records, the Huntsman Cancer Registry, and routinely collected PRO questionnaires. We assessed the association between DM in patients with cancer and PRO scores for anxiety, depression, fatigue, pain interference, and physical function using multiple linear regression and t‐tests.

**Results:**

The final cohort comprised 3512 patients with cancer, with a mean age of 57.8 years at cancer diagnosis. Of all patients, 49.1% (*n* = 1724) were female, with 82.0% (*n* = 2879) patients reporting PROs at least at one time point. Compared with patients who responded, nonresponders were more often female (*p* = 0.0035), less frequently non‐Hispanic White (*p* = 0.0058), and had a higher BMI (*p* = 0.0759). Patients with cancer and diabetes had worse PRO scores for anxiety (*p* = 0.0003), depression (*p* < 0.0001), fatigue (*p* < 0.0001), pain interference (*p* < 0.0001), and physical function (*p* < 0.0001) compared to patients with cancer without diabetes. Significant associations between diabetes and PRO scores were observed for anxiety (*β* ± SE: 1.27 ± 0.48; *p* = 0.0076), depression (*β* ± SE: 1.46 ± 0.45; *p* = 0.0011), fatigue (*β* ± SE: 2.11 ± 0.52; *p* < 0.0001), pain interference (*β* ± SE: 1.42 ± 0.50; *p* = 0.0046), and physical function (*β* ± SE: −2.74 ± 0.48; *p* < 0.0001).

**Conclusions:**

The results of this study suggest that patients with cancer and diabetes may be at greater risk for anxiety, depression, fatigue, higher pain interference, and reduced physical function. Strengthening diabetes management is imperative to address the negative impact of diabetes on PROs. In particular, this may be true for patients with skin, breast, prostate, and kidney cancer.

## 1 INTRODUCTION

Worldwide, cancer and diabetes are two of the most common diseases. In the United States (US), approximately 16.9 million individuals, or 5% of the population, are estimated to have cancer. Simultaneously, diabetes mellitus (diabetes) affects 30.3 million people, constituting 9.4% of the US population. Current studies have indicated that patients with cancer have an increased risk of developing diabetes,^1^ and overall, every third patient with cancer is diagnosed with diabetes.^2^

Studies exploring the connection between cancer and diabetes have traditionally focused on diabetes as a potential contributor to the development of various cancer types.^3^ Nevertheless, a more recent analysis has suggested that this association might work both ways, as the study revealed that cancer is a significant risk factor for the development of diabetes.^4^ There is mounting evidence that certain cancer therapies (e.g., chemotherapy and steroids) play an important role in this context.^5^, ^6^, ^7^, ^8^ More in detail, previous research has indicated that, in particular, systemic therapy is linked to diabetes by interfering with insulin secretion (e.g., immune checkpoint inhibitors),^9^ reducing insulin sensitivity (e.g., mTOR inhibitors),^10^ or both (e.g., selective estrogen receptor modulators).^11^ In addition, glucocorticoids, either used for cancer treatment or to treat side effects of therapy^12^ can also trigger new‐onset diabetes.

However, the simultaneous diagnosis of cancer and diabetes has far‐reaching consequences for the affected patients. With respect to patients with cancer, diabetes is associated with higher rates of complications,^13^, ^14^ increased rates of infections,^15^ and increased recurrence and mortality rates.^16^, ^17^, ^18^ Emerging evidence also indicates that unmanaged diabetes may impact cancer treatment completion, decrease the intensity of cancer therapy, and increase the vulnerability of cancer patients with diabetes to infections and unplanned hospitalizations throughout their cancer treatment journey.^19^, ^20^, ^21^

Overall, diabetes is a common cause of non‐cancer mortality in patients with cancer.^22^, ^23^ A current study has shown that in patients with prostate, breast, or colorectal cancer Type 2 diabetes is associated with a higher (HR 1.23; 1.21–1.25) overall mortality.^24^ Regarding diabetes management, the simultaneous diagnosis of cancer is associated with decreased medication adherence,^25^, ^26^ insufficient glycemic control,^27^ and often providers and patients focus on cancer treatment and diabetes treatment is a lower priority.^28^, ^29^

Over the last decade, patient‐reported outcomes (PROs) have become an integral part of clinical practice. PROs have proven to be valuable tools for evaluating the quality of care and the assessment of subjective health status.^30^, ^31^, ^32^ In patients with cancer, PROs can help to improve symptom management,^33^ to identify psychosocial problems,^34^ to facilitate patient‐centered care,^35^ to support patient–provider communication,^36^, ^37^, ^38^ and to improve clinical outcomes.^39^

However, evidence on the association of diabetes with PROs in patients with cancer remains scarce. Existing research is considerably heterogeneous and mainly focused on health‐related quality of life.^40^, ^41^, ^42^ The impact of diabetes on other PROs, such as fatigue or physical function, is understudied in patients with cancer.^43^ Also, prior studies are often based on one specific cancer and/or are lacking a comprehensible characterization of diabetes. Studies are usually based on ICD‐codes and do not include clinical data (e.g., hemoglobin A1c) to diagnose diabetes. In contrast, this study aimed to evaluate the association of diabetes with routinely collected PROs in patients with cancer, using a large, well‐characterized, prospective real‐world cohort of patients with cancer.

## 2 METHODS

### 2.1 Design and population

This cross‐sectional cohort study utilized the Total Cancer Care (TCC) protocol at the University of Utah (UofU) Huntsman Cancer Institute (HCI). Participants enrolled under this protocol agree to be followed up and to have their medical records obtained for research purposes, including cancer occurring before enrollment. The study population at the time of this project included *n* = 5865 individuals, men and women, who enrolled in TCC between July 1, 2016, and July 31, 2018. All participants have provided written informed consent. The HCI‐TCC protocol was approved by the University of Utah (UofU) Institutional Review Board (IRB #89989).

### 2.2 Data collection

Patients in this study were identified through the TCC Cancer Clinical Research (CCR) database. The CCR contains study information (e.g., enrollment date and unique patient identifier) and is linked with related research and clinical information systems at HCI. For this analysis, we included data from the Huntsman Cancer Registry and the University of Utah Health Enterprise Data Warehouse. Data from both sources (Table S1) were available for the time between January 2000 and July 2019 (for patients recruited between July 2016 and July 2018), allowing at least one to 3 years of follow‐up for each patient.

### 2.3 Inclusion/exclusion criteria

**FIGURE 1:**
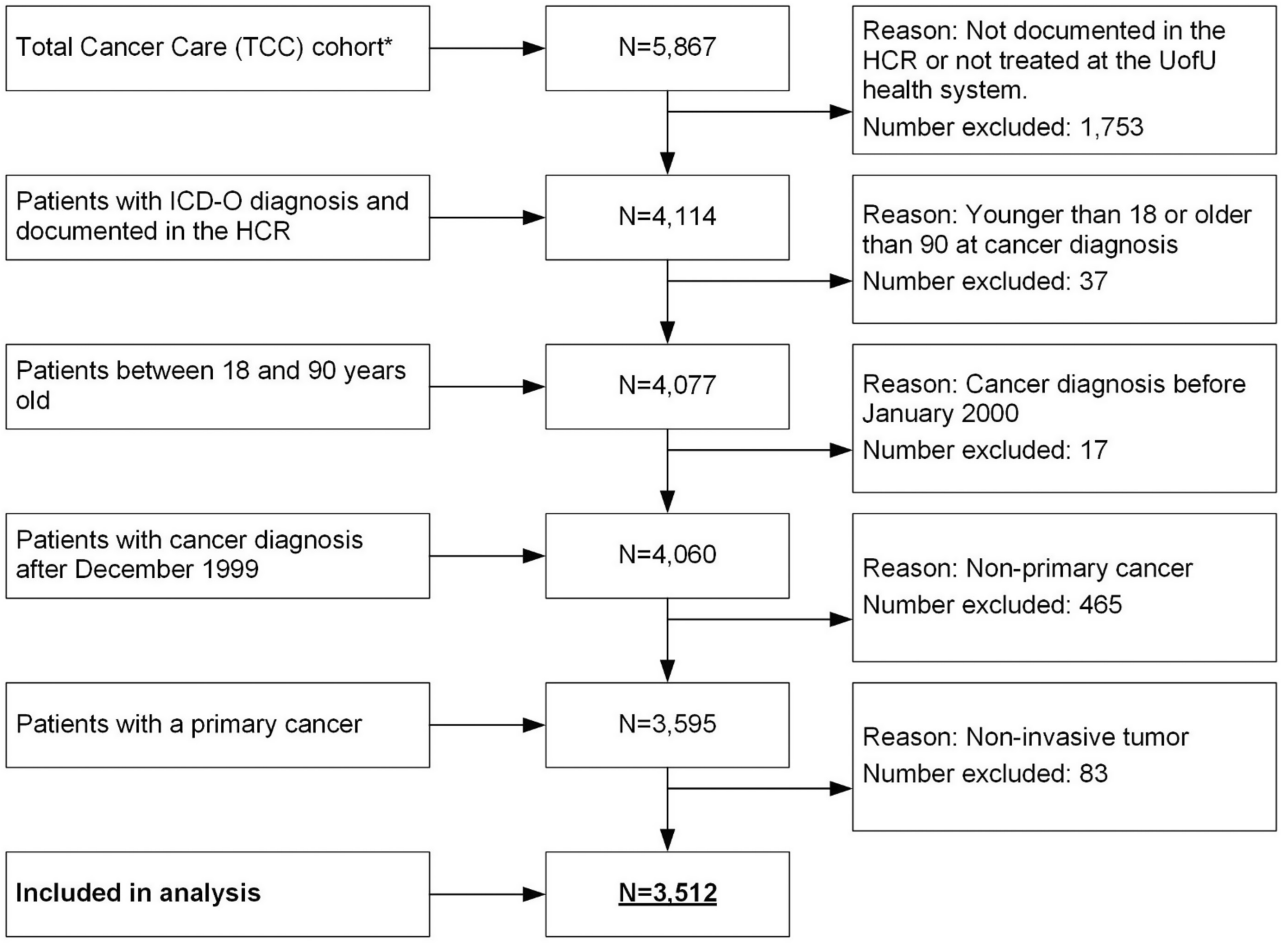
Flowchart of patient cohort.

Participant selection criteria included: age 18+ years, ICD‐O (International Classification of Diseases for Oncology) diagnosis, pathologically confirmed first primary invasive cancer, treated at the HCI between 2016 and 2019, and one or more reported PRO scores. Participants were excluded from the analysis if they did not have a documented ICD‐O diagnosis in the HCR, were not treated in the UofU Health system (HCR ‘class of case’ numbers: 30, 31, 33, 35, 37, 38, 40–43, 49, 99), were not diagnosed between 01/2000 and 12/2018, and were not between 18 and 90 years old at cancer diagnosis or the cancer was not primary or not invasive (Figure 1).

### 2.4 Cancer characteristics

The determination of the tumor behavior was based on ICD‐O morphology codes. Only patients with a tumor behavior code “3” (malignant, primary site) were included in this analysis. The determination of whether a tumor was the first tumor was assessed based on the sequence number, which is available from the cancer registry. In this analysis, we included patients with the sequence number “0” (first and only tumor) and “1” (first of more than one tumors). The onset of cancer was defined as the date of the first ICD‐O diagnosis documented in the Huntsman Cancer Registry (HCR), even if this date was prior to TCC enrollment. The definition of tumor sites was based on the SEER Site Recode of ICD‐O codes (ICD‐O‐3/WHO 2008 Definition).^44^

### 2.5 Determination of diabetes

The criteria for the determination of diabetes were based on the guidelines of the American Diabetes Association (ADA).^44^ The onset of diabetes was defined as the date of the first qualifying ICD code (ICD‐9250.xx, ICD‐10 E8‐E13), and/or laboratory results (hemoglobin A1c (HbA1c) >6.4%, fasting plasma glucose (FPG) >125 mg/dL, blood glucose (BG) >199), and/or prescription for insulin. If the determination of diabetes was based solely on a laboratory result, a second test, at least 3 days after the initial test, was required to confirm the diagnosis.

Diabetes status was classified into two categories: prevalent diabetes at cancer diagnosis and incident diabetes after cancer diagnosis. Diabetes at cancer diagnosis includes all patients who had diabetes at cancer diagnosis or were diagnosed with diabetes within 15 days after cancer diagnosis. Diabetes after cancer diagnosis is defined as developing diabetes at least 16 days after cancer diagnosis.

### 2.6 Diabetes timing and prevalence

The prevalence of diabetes in this real‐world cohort is high. At the end of the observation period (median follow‐up 28 months), the prevalence of diabetes was 32.6% (95% CI: 31.1‐34.2). However, at cancer diagnosis, in the prevaence of diabetes was 12.2% (95% CI: 11.2–13.3), and 1 year after cancer diagnosis was 25.0% (95% CI: 23.6–26.4), respectively. More information is provided elsewhere.^2^

### 2.7 Measures

#### 2.7.1 Patient‐reported outcome (PRO) measures

The University of Utah Health System (UofU Health) implemented standardized PROs in 2016. The core assessment is based on the PROMIS (Patient‐Reported Outcomes Measurement Information System) measures^45^ and is completed by patients at least annually and no more often than weekly via tablet or email,^46^ through computerized adaptive testing (CAT). By utilizing CAT, succeeding questions are based on preceding questions, which decreases the number of questions and the burden on the patient.^47^ Outcomes are reported by using standardized *T*‐scores (1–100) and are calibrated to a mean *T*‐score of 50 specific to the US general population with a standard deviation of 10.^48^, ^49^

The PROMIS measures included in this study are anxiety, depression, fatigue, pain interference, and physical function. The PROMIS measure for anxiety concentrates on fear, anxious misery, and arousal. for depression on negative mood, negative views of the self, and negative social cognition as well as for fatigue on subjective feelings of tiredness and the sustained sense of exhaustion linked to daily activities and social roles. In addition, pain interference evaluates self‐reported consequences of pain on relevant aspects of one's life as well as physical functioning and is measured as self‐reporting capabilities compared with the actual performance of physical activities.^45^

The population‐based estimates for mean scores across patients with cancer in the United States are 49.2 (0.2) for anxiety, 48.5 (0.2 SD) for depression, 52.2 (SD 0.2) for fatigue, 52.4 (SD 0.2) for pain interference, and 44.8 (SD 0.2) for physical function.^50^ All of the five PROMIS measures have been validated for patients with cancer.^51^, ^52^, ^53^, ^54^, ^55^ For anxiety, depression, fatigue, and pain interference, higher scores indicate worse functioning or an increase in symptoms. For physical function, higher scores indicate better functioning or a decrease in symptoms.

To improve the interpretability of PROs, minimally important differences (MID) can be utilized. MIDs reflect the difference in PRO scores that is large enough to have consequences for a patient's treatment or care. In patients with cancer, recommended *T*‐score MID ranges are for anxiety and depression 3.0–4.5, for fatigue 2.5–4.5, as well as for pain interference and physical function 4.0–6.0.^56^

#### 2.7.2 Covariates

Covariates were selected based on the properties of confounding factors. For example, all covariates are risk factors for decreased PROs and have associations with exposure (diabetes vs. no diabetes). Also, the covariates are not mediators between the exposure and included PROs.

More in detail, sex was defined as a biological variable to describe biological differences and influences when comparing men and women. Consequently, sex was binary coded as masculine or feminine rather than gender, which might exist on a spectrum and is most commonly used to indicate social or psychological differences between men, women, and other genders.^57^ Other covariates included were age at cancer diagnosis (<50 years, 50–64 years, 65–79 years, 80+ years); race and ethnicity (non‐Hispanic white, non‐Hispanic black; non‐Hispanic Asian; non‐Hispanic other; Hispanic), BMI (<18.50: underweight, 18.50–24.99: normal weight, 25.00–29.99: overweight, 30.00+: obese), population (rural, non‐rural, other), state of residence (Utah, Idaho, Wyoming, Nevada, Montana, other), cancer stage (stage 1, stage II, stage III, stage IV), Cancer treatment (Surgery, chemotherapy, radiation, hormone therapy, immunotherapy), and primary cancer (one primary cancer, multiple primary cancer).

### 2.8 Statistical analysis

Clinicodemographic characteristics were examined by diabetes status (no diabetes versus diabetes). Total counts and percentages among the diabetes status groups are displayed for categorical variables as well as means and standard deviations (SD) for continuous variables. Differences in clinicodemographic characteristics by diabetes status were compared using chi‐squared tests for categorical variables and one‐way ANOVA for continuous variables. PRO measures were assessed using the means and SD of last documented PRO scores after cancer diagnosis.

Multiple linear regression models (adjusted/unadjusted) were used to evaluate the association between diabetes status and overall PRO scores, with t‐tests comparing the differences. PROs were adjusted for age at cancer diagnosis, sex, race/ethnicity, BMI, Charlson comorbidity index (CCI), marital status, smoking status, cancer stage at diagnosis, and cancer type. To not reduce the sample size, missing values were not excluded for those regression models. However, we conducted separate analyses, one that included missing values and another that excluded missing values. We reported the *p*‐values and relevant results where appropriate. All statistical tests were two‐sided, and *p*‐values <0.05 were considered as statistically significant.

## 3 RESULTS

### 3.1 Description of the cohort

The HCI‐TCC‐diabetes cohort (Figure 1) comprised a total of 3512 patients with cancer (median follow‐up 28 months), with a mean age of 57.8 years at cancer diagnosis. Of all patients, 49.1% (*n* = 1724) were female, 90.5% (*n* = 3177) were non‐Hispanic White, 60.4% (*n* = 2122) were overweight or obese, and 70.2% (*n* = 2464) were Utah residents. With respect to tumor sites, cancers of the breast (11.7%; *n* = 410) and prostate (12.1%; *n* = 425) were the most common cancer diagnoses in this cohort (Table S3A). Regarding cancer severity and treatment, 31.2% (*n* = 1096) of all patients were diagnosed with stage III/IV cancer, 31.3% (*n* = 2957) had undergone surgery, and 15.4% (*n* = 1458) had undergone chemotherapy, 31.1% (*n* = 2938) of patients were treated with glucocorticoids (Table 1). Compared to patients without diabetes, patients with diabetes were older (*p* < 0.0001), more often male (*p* = 0.0173), more often Hispanic (*p* < 0.0001), and had a higher BMI (*p* < 0.0001) (Table 1).

**TABLE 1:** Patients characteristics by diabetes status among patients with cancer (*n* = 3512).^a^

Patients characteristics	Patients	No diabetes	Diabetes	*p*‐value
Study population *n* (%)	3512 (100)	2367 (67.4)	1145 (32.6)	
Age (years) mean (SD)^b^	57.8 (14.0)	56.6 (14.2)	60.4 (13.1)	<.0001^1^
Age categorized *n* (%)^b^				<.0001^1^
<50 years	854 (24.3)	648 (27.4)	206 (18.0)	
50–64 years	1440 (41.0)	977 (41.3)	463 (40.4)	
65–79 years	1091 (31.1)	668 (28.2)	423 (36.9)	
80+ years	127 (3.6)	74 (3.1)	53 (4.6)	
Sex *n* (%)				0.0173^1^
Female	1724 (49.1)	1195 (50.5)	529 (46.2)	
Male	1788 (50.9)	1172 (49.5)	616 (53.8)	
Race and ethnicity *n* (%)				<.0001^2^
Non‐Hispanic White	3177 (90.5)	2171 (91.7)	1006 (87.9)	
Non‐Hispanic Black	18 (0.5)	16 (0.7)	2 (0.2)	
Non‐Hispanic Asian	38 (1.1)	23 (1.0)	15 (1.3)	
Non‐Hispanic Other^c^	142 (4.0)	88 (3.7)	54 (4.7)	
Hispanic	137 (3.9)	69 (2.9)	68 (5.9)	
BMI kg/m^2^ mean (SD)^d^	29.1 (6.6)	28.0 (5.8)	31.3 (7.7)	<0.0001
BMI kg/m^2^ category *n* (%)^d^				<.0001^2^
Underweight (<18.5)	33 (0.9)	24 (1.0)	9 (0.8)	
Normal (18.5–24.99)	814 (23.2)	629 (26.6)	185 (16.2)	
Overweight (25.0–29.99)	1004 (28.6)	723 (30.5)	281 (24.5)	
Obese (≥30)	1118 (31.8)	636 (26.9)	482 (42.1)	
Unknown	543 (15.5)	355 (15.0)	188 (16.4)	
Population *n* (%)^e^				0.4340^2^
Rural	1125 (32.0)	763 (32.2)	362 (31.6)	
Non‐Rural	2357 (67.1)	1587 (67.1)	770 (67.2)	
Unknown	30 (0.9)	17 (0.7)	13 (1.1)	
State of residence *n* (%)^f^				0.0530^2^
Utah	2464 (70.2)	1646 (69.5)	818 (71.4)	
Idaho	425 (12.1)	279 (11.8)	146 (12.8)	
Wyoming	274 (7.8)	114 (4.8)	86 (7.5)	
Nevada	168 (4.8)	188 (7.9)	54 (4.7)	
Other	181 (5.2)	140 (5.9)	41 (3.6)	
Cancer stage *n* (%)				<.0001^3^
Stage I	872 (24.8)	646 (27.3)	226 (19.7)	
Stage II	638 (18.2)	430 (18.2)	208 (18.2)	
Stage III	553 (15.7)	386 (16.3)	167 (14.6)	
Stage IV	543 (15.5)	344 (14.5)	199 (17.4)	
Unknown/ Not Applicable^g^	906 (25.8)	561 (23.7)	345 (30.1)	
Cancer treatment *n* (%)				0.0932^1^
Surgery	2957 (31.3)	2012 (31.8)	945 (30.1)	
Chemotherapy	1458 (15.4)	910 (14.4)	548 (17.5)	
Radiation	1035 (10.9)	686 (10.9)	349 (11.1)	
Hormone therapy	731 (7.7)	527 (8.3)	204 (6.5)	
Immunotherapy	339 (3.6)	217 (3.4)	122 (3.9)	
Glucocorticoids	2938 (31.1)	1966 (31.1)	972 (31.0)	
Cancer sequence number *n* (%)				0.0017^1^
00—only one primary cancer	3084 (87.8)	2107 (89.0)	977 (85.3)	
01—multiple primary cancer	428 (12.2)	260 (11.0)	168 (14.7)	

Abbreviations: BMI, body mass index; *n*, number; SD, standard deviation.

^a^ Not all %s add up to 100 because of rounding decimal places.

^b^ At cancer diagnosis.

^c^ American Indian/Alaska Native, Hawaiian/Other Pacific Islander, Other, or Unknown;

^d^ Body Mass Index, at cancer diagnosis (90 days window before and after cancer diagnosis).

^e^ Determined from RUCA score on zip code.

^f^ Determined from last known residence.

^g^ Brain and nervous system cancers are not routinely staged;

^1^ No missing values;

^2^ *p*‐values same with/without missing values; *p*‐value is 0.7724 without missing values.

^3^ *p*‐value is 0.0002 without missing values.

### 3.2 Completion of PROs

Of all patients in this cohort, 82.0% (*n* = 2879) responded at least once, with 53.1% (*n* = 1866) responding at least at two points in time, and 34.1% (*n* = 1199) responding at least at three points in time to one of the PRO measures (anxiety, depression, fatigue, pain interference, and physical function) during the observation period.

### 3.3 Responder versus nonresponder

Compared with questionnaire responders, nonresponders were more often female (*p* = 0.0035), less often non‐Hispanic White (*p* = 0.0058), and had a higher BMI (*p* = 0.0759). With respect to cancer treatment, nonresponders were more often diagnosed with Stage I and less often Stage IV (*p* < 0.0001) as well as received less often chemotherapy (*p* < 0.0001; Table S2). The median time from cancer diagnosis to PRO documentation was 490 days for anxiety, 492 days for depression, and 497 days for fatigue, pain interference, and physical function.

### 3.4 PRO scores by diabetes status and time point for all tumor sites

**FIGURE 2:**
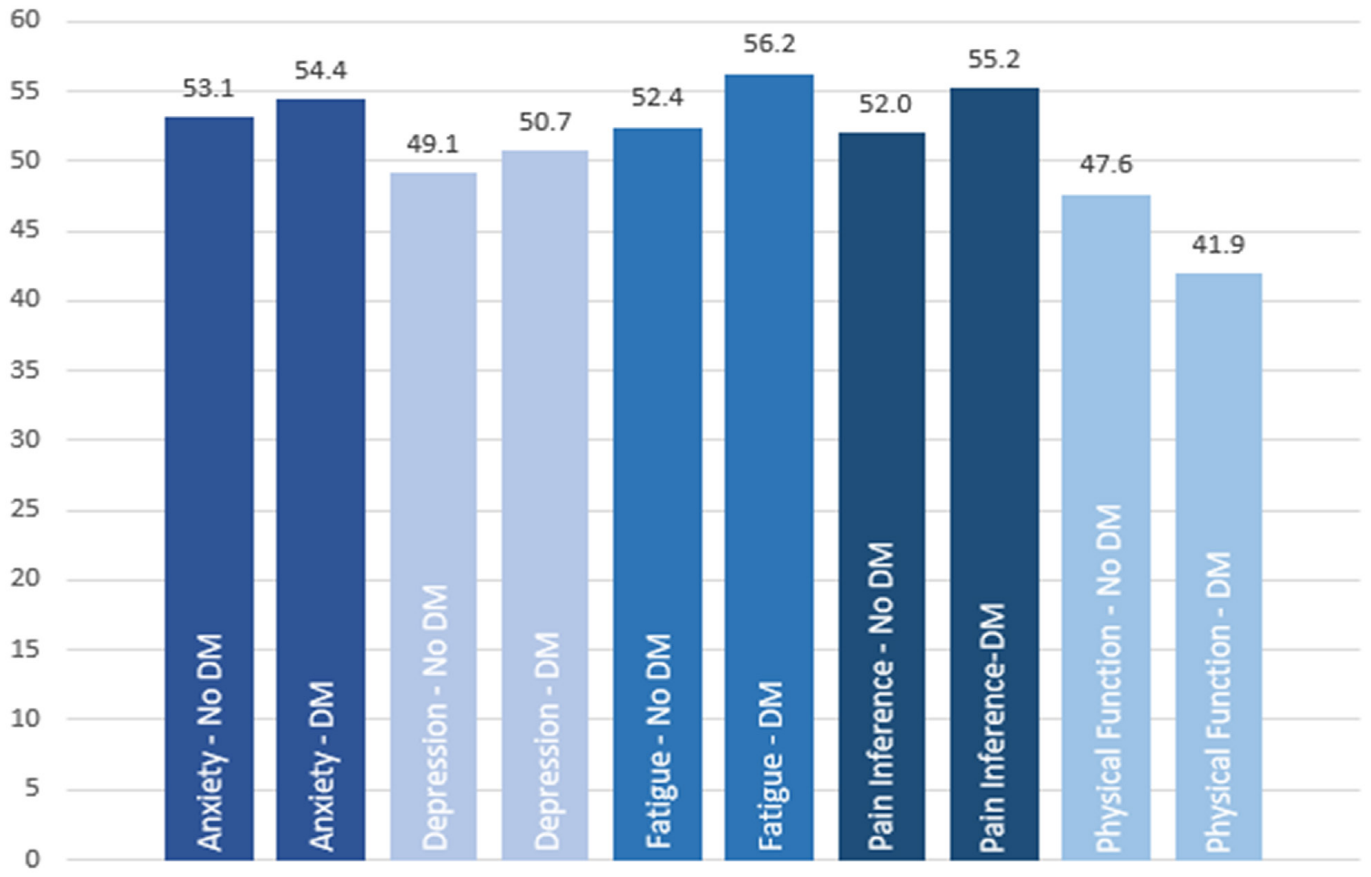
PRO Scores by DM Status.

Overall, patients with cancer and diabetes had worse PRO scores for anxiety (*p* = 0.0003), depression (*p* < 0.0001), fatigue (*p* < 0.0001), pain interference (*p* < 0.0001), and physical function (*p* < 0.0001) compared to patients without diabetes (Table 2). In particular, substantial differences existed between patients without diabetes and patients with diabetes for the mean PRO scores for fatigue (52.4 vs. 56.2), pain interference (52.0 vs. 55.2), and physical function (47.6 vs. 41.9). With respect to the onset of diabetes (before or at cancer diagnosis vs. after cancer diagnosis), no significant differences were observed between the PRO scores, except for fatigue (*p =* 0.14), pain interference (*p* = 0.0044), and physical function (*p* = 0.0002; Table 2, Figure 2).

**TABLE 2:** PRO scores by diabetes status and time point for all tumor sites.^c^

PROs	No‐DM	(*n* = 2367)	DM	(*n* = 1145)	*p*	DM at CADX	^a^ (*n* = 429)	DM after CADX^b^	(*n* = 716)	*p*
	*N* Res	Mean (SD)	*N* Res	Mean (SD)		N Res	Mean (SD)	*N* Res	Mean (SD)	
Anxiety	1895	53.1 (8.87)	948	54.4 (8.63)	0.0003*	323	54.5 (8.69)	625	54.3 (8.61)	0.673
Depression	1894	49.1 (8.27)	947	50.7 (8.45)	<0.0001*	323	51.4 (8.87)	624	50.4 (8.21)	0.0959
Fatigue	1901	52.4 (9.99)	949	56.2 (9.01)	<0.0001*	325	57.2 (9.08)	624	55.6 (8.93)	0.014*
Pain interference	1906	52.0 (9.25)	949	55.2 (8.98)	<0.0001*	325	56.4 (8.75)	624	54.6 (9.05)	0.0044*
Physical Function	1911	47.6 (9.99)	952	41.9 (8.74)	<0.0001*	327	40.5 (8.65)	625	42.7 (8.70)	0.0002*

^a^ Patients already had diabetes or diabetes was diagnosed at cancer diagnosis.

^b^ Patients developed diabetes after cancer diagnosis.

^c^ Unadjusted.

*Note*: Bold values indicates *P* < 0.05.

### 3.5 PRO scores by diabetes status for selected tumor sites

**FIGURE 3:**
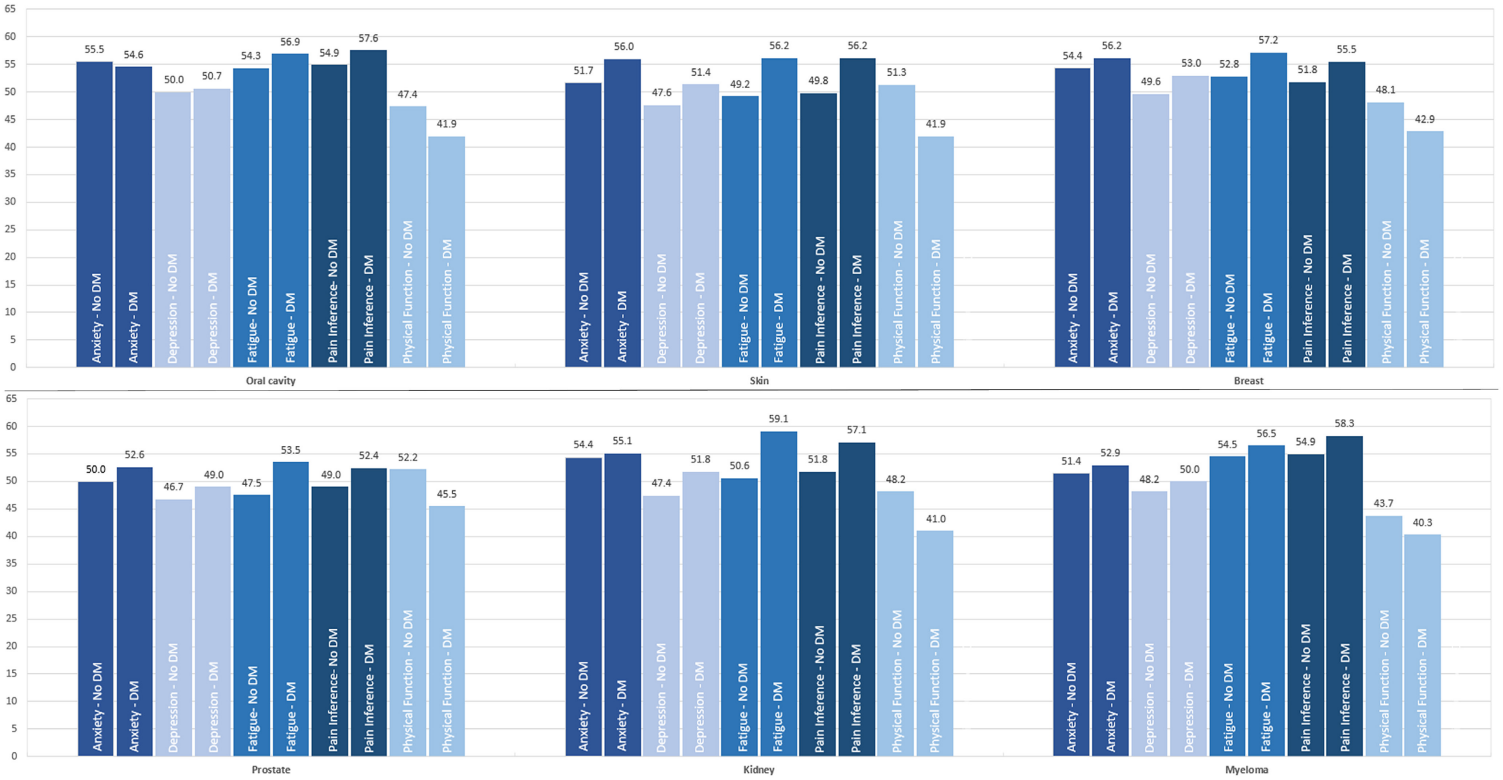
PRO Scores by DM Status and Cancer Type.

Differences in PRO scores between patient without diabetes and patients with diabetes varied with respect to the specific tumor site and the specific PRO measure. Statistically significant differences were observed, for example, in patients with breast cancer for depression (49.6 vs 53.0; *p* < 0.001), fatigue (52.8 vs. 57.2; *p* < 0.001), pain interference (51.8 vs. 55.5; *p* < 0.001), and physical function (48.1 vs. 42.9; *p* < 0.001) or in patients with prostate cancer for anxiety (50.0 vs. 52.6; *p* < 0.01), depression (46.7 vs. 49.0; *p* < 0.05), fatigue (47.5 vs. 53.5; *p* < 0.001), pain interference (49.0 vs. 52.4; *p* < 0.01), and physical function (52.2 vs. 45.5; *p* < 0.001) (Table 3, Figure 3). An overview for all cancer types is presented in the online appendix (Table S4A,B).

**TABLE 3:** PRO scores by diabetes status for selected tumor sites.^a^ ^,^ ^b^ ^,^ ^c^

Tumor site	*N*	Anxiety		Depression		Fatigue		Pain interference		Physical function	
		No‐DM	DM	No‐DM	DM	No‐DM	DM	No‐DM	DM	No‐DM	DM
		Mean (SD)	Mean (SD)	Mean (SD)	Mean (SD)	Mean (SD)	Mean (SD)	Mean (SD)	Mean (SD)	Mean (SD)	Mean (SD)
Oral cavity^d^	101	55.5 (8.46)	54.6 (7.95)	50.0 (6.82)	50.7 (7.21)	54.3 (9.93)	56.9 (7.28)	54.9 (9.24)	57.6 (8.01)	47.4 (9.13)	41.9 (7.19)* **
Skin	192	51.7 (9.82)	56.0 (8.59)**	47.6 (8.48)	51.4 (8.02)*	49.2 (9.91)	56.2 (9.99)* **	49.8 (9.5)	56.2 (9.62)* **	51.3 (10.12)	41.9 (9.07)* **
Breast	415	54.4 (8.78)	56.2 (8.37)	49.6 (8.02)	53.0 (8.25)***	52.8 (9.88)	57.2 (8.99)* **	51.8 (8.76)	55.5 (8.68)* **	48.1 (9.6)	42.9 (8.97)* **
Prostate	433	50.0 (7.34)	52.6 (7.76)**	46.7 (7.37)	49.0 (7.87)*	47.5 (9.39)	53.5 (8.79)* **	49.0 (8.38)	52.4 (8.57)**	52.2 (9.14)	45.5 (7.79)* **
Kidney^e^	64	54.4 (9.06)	55.1 (6.73)	47.4 (8.03)	51.8 (8.1)*	50.6 (11.52)	59.1 (7.63)* **	51.8 (9.33)	57.1 (8.19)*	48.2 (9.01)	41.0 (7.23)* **
Myeloma	174	51.4 (7.73)	52.9 (8.31)	48.2 (8.07)	50.0 (7.48)	54.5 (8.94)	56.5 (7.72)	54.9 (8.18)	58.3 (7.28)**	43.7 (8.2)	40.3 (8.04)* **

Abbreviation: Res, Responder.

^a^ At the end of observation period.

^b^ A complete overview for all tumor sites can be found in the online‐appendix;

^c^ Unadjusted.

^d^ And Pharynx.

^e^ And Renal Pelvis.

* *p* < 0.05.

** *p* < 0.01.

*** *p* < 0.001.

### 3.6 Association between clinicodemographics and PRO scores

Statistically significant associations between clinicodemographics and PRO scores were observed, for example, for age at cancer diagnosis (anxiety: *β* ± SE −0.06 ± 0.01, *p* < 0.0001; depressions: *β* ± SE −0.06 ± 0.01, *p* < 0.0001; physical function: *β* ± SE −0.12 ± 0.01, *p* < 0.0001), female sex (anxiety: *β* ± SE 2.96 ± 0.33, *p* < 0.0001; depressions: *β* ± SE 2.14 ± 0.31, *p* < 0.0001; fatigue: *β* ± SE 2.86 ± 0.37, *p* < 0.0001; pain interference: *β* ± SE 1.29 ± 0.35, *p* < 0.0002; physical function: *β* ± SE −2.64 ± 0.37, *p* < 0.0001), not being married (anxiety: *β* ± SE 1.85 ± 0.39, *p* < 0.0001; depressions: *β* ± SE 2.00 ± 0.37, *p* < 0.0001; fatigue: *β* ± SE 2.64 ± 0.43, *p* < 0.0001; pain interference: *β* ± SE 2.06 ± 0.41, *p* < 0.0001; physical function: *β* ± SE −2.83 ± 0.44, *p* < 0.0001) (Table S4).

### 3.7 Association of diabetes with PRO scores

Statistically significant associations between diabetes and PRO scores were observed for anxiety (*β* ± SE 1.26 ± 0.35; *p* = 0.0003), depression (*β* ± SE: 1.58 ± 0.33; *p* < 0.0001), fatigue (*β* ± SE: 3.78 ± 0.38; *p* < 0.0001), pain interference (*β* ± SE: 3.25 ± 0.36; *p* < 0.0001), and physical function (*β* ± SE: −5.72 ± 0.38; *p* < 0.0001). However, associations differed between female and male patients with cancer. Whereas the association was significant for all PRO measure in male patients with cancer, the association among female patients with cancer was only significant for fatigue, pain interference, and physical function (Table 4, Table S5).

**TABLE 4:** Association of diabetes with PRO scores (A: missing not included); (B: missing included as separate category).

PROs	All patients (*n* = 3512)				Women (*n* = 1724)				Men (*n* = 1788)			
	No‐DM	DM	*β* ± SE^a^	*p*	No‐DM	DM	*β* ± SE^b^	*p*	No‐DM	DM	*β* ± SE^b^	*p*
	*N*	*N*			N	N			*N*	*N*		
(A)												
Anxiety	1239	555	1.27 ± 0.48	0.0076*	633	276	1.173 ± 0.69	0.0914	606	279	1.085 ± 0.65	0.0974
Depression	1238	555	1.468 ± 0.45	0.0011*	633	276	1.127 ± 0.65	0.0825	605	279	1.645 ± 0.63	0.0087*
Fatigue	1244	554	2.107 ± 0.52	0.0001*	637	275	1.428 ± 0.74	0.0546*	607	279	2.613 ± 0.73	**0.0004**
Pain interference	1248	554	1.416 ± 0.5	0.0046*	640	275	0.883 ± 0.71	0.2115	608	279	1.788 ± 0.71	0.0120*
Physical function	1253	556	−2.747 ± 0.48	<0.0001*	643	276	−1.716 ± 0.68	**0.0117**	610	280	−3.676 ± 0.7	<0.0001*
(B)												
Anxiety	1895	948	0.911 ± 0.36	0.0120*	927	434	0.608 ± 0.55	0.2692	968	514	1.067 ± 0.48	0.0273*
Depression	1894	947	1.202 ± 0.35	0.0005*	927	433	0.629 ± 0.52	0.2264	967	514	1.592 ± 0.47	0.0006*
Fatigue	1901	949	2.456 ± 0.39	<0.0001*	933	433	1.384 ± 0.58	0.0178*	968	516	3.266 ± 0.53	<0.0001*
Pain interference	1906	949	1.805 ± 0.38	**<0.0001**	936	433	0.905 ± 0.57	0.1107	970	516	2.491 ± 0.51	<0.0001*
Physical function	1911	952	−3.333 ± 0.38	<0.0001*	939	435	−2.389 ± 0.54	<0.0001*	972	517	−4.055 ± 0.52	<0.0001*

^a^ Adjusted for age at cancer diagnosis, sex, BMI, CCI, marital status, smoking status, cancer stage at diagnosis, and cancer types.

^b^ Adjusted for age at cancer diagnosis, race/ethnicity, BMI, CCI, marital status, smoking status, cancer stage at diagnosis, and cancer type.

*Note*: Bold values indicates *P* < 0.05.

## 4 DISCUSSION

This large, prospective, cohort study evaluated the association of diabetes with routinely collected PROs in patients with cancer. Our analysis had three main results^1^: Overall, patients with cancer and diabetes had significantly lower PRO scores with respect to anxiety, depression, fatigue, pain interference, and physical function, compared to patients with cancer who were not diagnosed with diabetes.^2^ Not all cancer types were affected equally. Significantly lower PRO scores in patients with cancer and diabetes were observed in patients with skin, breast, and prostate cancer.^3^ Adjusted for sociodemographic and cancer characteristics, the results of this study suggest that patients with cancer and diabetes may be at greater risk for anxiety, depression, fatigue, higher pain interference, and reduced physical function.

These findings are novel. To the best of our knowledge, this is the first study systematically analyzing the association between diabetes and PROs in a large, well‐characterized real‐world cancer cohort. Previous studies were commonly focused on a single disease and/or a specific PRO. However, often the results of those studies pointed in the same direction. For example, the systematic review of Hammer and colleagues (2019) reported poorer physical function and greater fatigue in women with breast cancer and diabetes compared to women with breast cancer without diabetes.^58^ With respect to anxiety, the analysis from Han (2020) revealed that diabetes was a highly significant predictor in patients with gastric cancer.^59^ Previous research also showed that diabetes is an important risk factor for developing postoperative depression in patients with lung cancer.^60^ In addition, a current qualitative analysis by Pinheiro and colleagues (2023) indicates that the combination of worsening glycemic control and the burden of cancer treatment can lead to negative psychosocial consequences, such as anxiety and depression.^27^

This is problematic, as diabetes is very common in patients with cancer. As shown by our previous analysis, about 30% of patients across all cancer types suffer from diabetes, many of them developing diabetes after cancer diagnosis. However, diagnosing and managing diabetes in patients with cancer remains a challenge. Glycemic control is frequently insufficient^61^ and pre‐diabetes as well as diabetes is frequently undiagnosed in patients with cancer.^62^ In addition, the lack of collaboration and communication between providers (e.g., oncologists, diabetologists, and PCPs) and other health professions (e.g., nurses and nutritionists) can lead to poorly coordinated care, the duplication of clinical procedures, unnecessary admissions, and delayed access to services.^63^

### 4.1 Clinical implications

Despite this situation, strengthening diabetes management in patients with cancer is imperative to address the negative impact of diabetes on patient‐reported outcomes, such as anxiety, depression, fatigue, pain interference, and physical function. In particular, that is true for patients with skin, breast, prostate, and kidney cancer. However, diabetes management in patients with cancer remains underdeveloped in most health systems. At the moment, only in the United Kingdom (UK), a guideline for the management of glycemic control in patients with cancer is available.^63^, ^64^

The guideline summarizes available evidence and is recommending pathways to facilitate the management of patients diagnosed with cancer and diabetes. For example, the guideline suggests a baseline HbA1c and a plasma glucose check before commencing anti‐cancer therapy or steroids, monitoring of random plasma glucose at each treatment visit, patient education, and collaborating with the diabetes team, for example, for support in titrating insulin.^64^ However, in the absence of specific guidelines, diabetes should be managed pragmatically, to ensure the patient is kept asymptomatic and at low risk of acute decompensation. Proactive management of treatment‐induced hyperglycemia and diabetes may help reduce large fluctuations in glucose levels. However, whether improving diabetes management in patients with cancer also improves PROs remains uncertain. To date, there is no evidence from randomized clinical trials (RCT) to investigate whether treating hyperglycemia in patients with cancer improves health‐related outcomes.^65^

Overall, routinely implemented PROs can help to monitor the impact of cancer treatment on patients' well‐being. Treatments such as surgery, radiotherapy, and chemotherapy often cause acute and late side effects, not only related to diabetes.^66^ Previous studies have shown that PROs are more accurate in capturing patient symptoms compared with the assessment of physicians^67^ and hence are essential for the evaluation of symptoms in cancer survivors.^68^ The implementation of PROs in clinical care can not only strengthen the management of cancer and treatment‐related effects,^69^ but also improve the quality of life in patients with cancer.^70^

### 4.2 Strengths and limitations

This study has several strengths. The basis for this analysis is the HCI‐TCC‐DM cohort, a large real‐world cancer patient population from the Western United States (Utah, Idaho, Wyoming, Nevada, and Montana). Our population was restricted to patients diagnosed with a pathologically confirmed first primary invasive cancer. Through the linkage of the HCI‐TCC clinical data repository, the Huntsman Cancer Registry and the University of Utah Health Enterprise Data Warehouse (EDW), a wealth of clinical and study‐related data was available for this analysis. In particular, to link routinely collected PROs with clinical routine data as well as data from the cancer registry is highly innovative.

This study also has several limitations. The ADA guidelines have not been specifically developed for patients with cancer. Whether elevated laboratory results, even if they were 3 days apart, were diagnostic for diabetes, cannot be determined conclusively. However, the diagnosis of DM was solely determined by laboratory values only in 7.9% of all patients with DM (*n* = 1145). Also, because the PROs used for this analysis are routinely collected, the number of available PROs and the time points where the PROs were filled out are varying. To address this situation, we used the last documented PRO for each patient.

## 5 CONCLUSION

The results of this study suggest that patients with cancer and diabetes may be at greater risk for anxiety, depression, fatigue, higher pain interference, and reduced physical function. Strengthening diabetes management is imperative to address the negative impact of diabetes on patient‐reported outcomes. In particular, this may be true for patients with skin, breast, prostate, and kidney cancer.

## AUTHOR CONTRIBUTIONS

**Dominik J. Ose:** Conceptualization (lead); data curation (equal); formal analysis (equal); funding acquisition (equal); investigation (lead); methodology (equal); writing – original draft (lead). **Emmanuel Adediran:** Project administration (equal); resources (equal). **Bayarmaa Mark:** Formal analysis (equal). **Krista Ocier:** Formal analysis (lead); methodology (lead); writing – review and editing (equal). **William A. Dunson JR:** Conceptualization (supporting); supervision (equal); validation (equal); writing – review and editing (equal). **Cindy Turner:** Conceptualization (equal); data curation (equal); project administration (lead); writing – review and editing (equal). **Belinda Taylor:** Data curation (equal); methodology (equal); supervision (equal); writing – review and editing (equal). **Kim Svoboda:** Data curation (equal); project administration (equal); supervision (equal); writing – review and editing (equal). **Andrew R. Post:** Data curation (equal); methodology (equal); supervision (equal); writing – review and editing (equal). **Jennifer Leiser:** Supervision (equal); writing – review and editing (equal). **Howard Colman:** Supervision (equal); validation (equal); writing – review and editing (equal). **Cornelia M. Ulrich:** Conceptualization (equal); funding acquisition (equal); supervision (equal); writing – review and editing (equal). **Mia Hashibe:** Conceptualization (equal); methodology (equal); supervision (lead); validation (lead); writing – review and editing (lead).

## FUNDING INFORMATION

This work was supported by a grant from the ‘Driving out Diabetes: A Larry H. Miller Family Wellness Initiative’. This work was also supported by grants from the National Institutes of Health/National Cancer Institute (U01 CA206110, R01 CA189184, and R01 CA207371 to Ulrich) and the Huntsman Cancer Foundation, and it *utilized the Research Informatics Shared Resource at Huntsman Cancer Institute under Award Number P30CA042014*. The content is solely the responsibility of the authors and does not necessarily represent the official views of the NIH.

## CONFLICT OF INTEREST STATEMENT

Dr. Ulrich has as cancer center director oversight over research funded by several pharmaceutical companies but has not received funding directly herself. The remaining authors have disclosed that they have not received any financial consideration from any person or organization to support the preparation, analysis, results, or discussion of this article.

## Supporting information


Data S1.

## Data Availability

The data that support the findings of this study are available from the corresponding author upon reasonable request.
