# Supplementary material for: The association of diabetes mellitus and routinely collected patient‐reported outcomes in patients with cancer. A real‐world cohort study
Source: Cancer Med. 2024 Oct 24;13(20):e70246. doi: 10.1002/cam4.70246 (PMC11500209; doi:10.1002/cam4.70246)
Supplement: Supplementary file 1 — Data S1. [file CAM4-13-e70246-s001.docx]

**Supplementary Online Content**

**eTable 1:** Data description

**eTable 2:** Comparing clinicodemographic characteristics between responder and non-responder

**eTable 3a:** Mean scores for Anxiety and Depression by diabetes status and tumor site

**eTable 3b:** Mean scores for Fatigue, Pain interference and Physical Function by diabetes status and tumor site

**eTable 4:** Association between clinicodemographics and PRO scores (unadjusted)

**eTable 5:** Association of diabetes on patient reported outcomes in patients with cancer (unadjusted)

**eTable 1:** Data description

| **Type and name** | | **Source** | **Description** | **Coding (example)** |
| --- | --- | --- | --- | --- |
| Patient identifier | | TCC | Unique number assigned by the EMR for patient identification across the UofU health system | 089761 |
| Demographics | |  |  |  |
|  | Sex | EDW | Patient-reported | Male; female |
|  | BMI (mean) | EDW | Calculated, based on  clinical measures | 25.3 |
|  | BMI groups | EDW | Calculated, based on  clinical measures | Underweight <18.5; Healthy weight 18.5 to 24.9; Overweight 25.0 to 29.9; Obese 30+ |
|  | Race and ethnicity | EDW | Patient-reported | Non-Hispanic white; Non-Hispanic black; Non-Hispanic Asian; Non-Hispanic other; Hispanic |
|  | State of residence | EDW | Patient-reported | Utah; Idaho; Wyoming; Nevada; other |
|  | Population | EDW | Calculated, based on  RUCA codes using ZIP | NON rural, rural |
| Dates | |  |  |  |
|  | Date of birth (DOB) | EDW | Patient-reported | 04/12/1978 |
|  | Diabetes / pre-diabetes diagnosis* | EDW | Date of qualifying ICD diagnosis, lab value or insulin prescription | 02/03/2017 |
|  | Cancer diagnosis | HCR | First cancer diagnosis documented in HCR | 01/03/2017 |
| Definition of DM/PRE-DM | |  |  |  |
|  | ICD-codes | EDW | ICD-codes documented in the EDW | Pre-Diabetes (ICD 9: 790.xx; ICD 10: R73.xx); Diabetes (ICD 9: 250.xx; ICD 10: E8 to E13) |
|  | Elevated glucose | EDW | Calculated, based on lab  tests | Pre-Diabetes (A1C (%) 5.7-6.4; Fasting plasma glucose (mg/dl) 100-125; Blood glucose (mg/dl) 140-199); Diabetes A1C (%) ≥ 6.5; Fasting plasma glucose (mg/ dl) ≥ 126; Blood glucose (mg/dl) ≥ 200) |
|  | Insulin prescription | EDW | Calculated, based on documented prescriptions | No; Yes |
| Cancer characteristics | |  |  |  |
|  | ICD-O diagnosis*** | HCR | Documented ICD-O Histo-code and Site-code | 8120/3; C67.9 |
|  | Stage | HCR | Documented “Best Dominant Stage” | 1a |
|  | Age at diagnosis | HCR | Calculated, based on DOB and cancer diagnosis Date | 48 years |
|  | Treatment | HCR | Abstracted form HCR trough certified tumor registrars | Surgery, chemotherapy, radiation, hormone therapy, immunotherapy |
|  | Sequence Number** | HCR | Sequence of tumor | 01 |

* To confirm diagnosis, 2+ laboratory test results more than two days apart were required if no other indication of diagnosis was given (ICD code, insulin prescription); ** Restricted to 0 or 1 (primary cancers); *** Restricted /3 (invasive cancer, primary site): HCR: Huntsman Cancer Institute; EDW: Enterprise Data Warehouse

**eTable 2:** Comparing Patients Characteristics between Responder and Non-Responder (n=3,512)

| **Patients Characteristics** | **Non-Responder**^1^ | **Responder** | **p-value** |
| --- | --- | --- | --- |
| **Population n (%)** | 633 (18.0) | 2,879 (82.0) | <.0001 |
| **Age (years) mean (SD)^b^** | 58.3 (15.2) | 57.7 (13.7) | 0.3667 |
| **Age categorized n (%)^b^** |  |  | <.0001 |
| <50 years | 167 (26.4) | 687 (23.9) |  |
| 50 to 64 years | 240 (37.9) | 1,200 (41.7) |  |
| 65 to 79 years | 181 (28.6) | 910 (31.6) |  |
| 80+ years | 45 (7.1) | 82 (2.9) |  |
| **Sex n (%)** |  |  | 0.0035 |
| Female | 344 (54.3) | 1,380 (47.9) |  |
| Male | 289 (45.7) | 1,499 (52.1) |  |
| **Race and Ethnicity n (%)** |  |  | 0.0058 |
| Non-Hispanic White | 533 (87.4) | 2,624 (91.1) |  |
| Non-Hispanic Black | 7 (1.1) | 11 (0.4) |  |
| Non-Hispanic Asian | 6 (1.0) | 32 (1.1) |  |
| Non-Hispanic Other^c^ | 38 (6.0) | 104 (3.6) |  |
| Hispanic | 29 (4.6) | 108 (3.8) |  |
| **BMI kg/m^2^ mean (SD)^d^** | 29.3 (7.0) | 29.0 (6.6) | 0.4597 |
| **BMI kg/m^2^ category n (%)^d^** |  |  | 0.0759^3^ |
| Underweight (<18.5) | 10 (1.6) | 23 (0.8) |  |
| Normal (18.5 - 24.99) | 157 (24.8) | 657 (22.8) |  |
| Overweight (25.0 - 29.99) | 161 (25.4) | 843 (29.3) |  |
| Obese (>=30) | 214 (33.8) | 904 (31.4) |  |
| Unknown | 91 (14.4) | 452 (15.7) |  |
| **Population n (%)^e^** |  |  | 0.882^4^ |
| Rural | 198 (31.3) | 927 (32.2) |  |
| Non-Rural | 430 (67.9) | 1,927 (66.9) |  |
| Unknown | 5 (0.8) | 25 (0.9) |  |
| **State of Residence n (%)^f^** |  |  | 0.0172 |
| Utah | 422 (66.7) | 2,042 (70.9) |  |
| Idaho | 90 (14.2) | 335 (11.6) |  |
| Wyoming | 43 (6.8) | 231 (8.0) |  |
| Nevada | 32 (5.1) | 136 (4.7) |  |
| Other | 46 (7.3) | 135 (4.7) |  |
| **Cancer Stage n (%)** |  |  | <.0001^5^ |
| Stage I | 230 (36.3) | 642 (22.3) |  |
| Stage II | 118 (18.6) | 520 (18.1) |  |
| Stage III | 81 (12.8) | 472 (16.4) |  |
| Stage IV | 45 (7.1) | 498 (17.3) |  |
| Unknown/ Not Applicable^g^ | 159 (25.1) | 747 (26.0) |  |
| **Cancer treatment n (%)** |  |  |  |
| Surgery | 568 (89.7) | 2,389 (83.0) | <.0001 |
| Chemotherapy | 143 (22.6) | 1,315 (45.7) | <.0001 |
| Radiation | 116 (18.3) | 919 (31.9) | <.0001 |
| Hormone therapy | 88 (13.9) | 643 (22.3) | <.0001 |
| Immunotherapy | 25 (4.0) | 314 (10.9) | <.0001 |
| Glucocorticoids | 490 (77.4) | 2,448 (85.0) | <.0001 |
| **Cancer Sequence Number n (%)** |  |  | 0.0005 |
| 00 – only one primary cancer | 582 (91.9) | 2,502 (86.9) |  |
| 01 – multiple primary cancer | 51 (8.1) | 377 (13.1) |  |

^a^ Not all %s add up to 100 because of rounding decimal places; ^b^ At cancer diagnosis; ^c^ American Indian/Alaska Native, Hawaiian/Other Pacific Islander, Other, or Unknown; ^d^ Body Mass Index, at cancer diagnosis (90 days window before and after cancer diagnosis); ^e^ Determined from RUCA score on zip code; ^f^ Determined from last known residence; ^g^ Brain and nervous system cancers are not routinely staged; n=number; SD=standard deviation; BMI=body mass index^1^ People who did not have any PRO or people only had PRO date only before and same day as CA-DX: ^2^ PROs available at least at one time point during the observation period: ^3^P-values=0.0528 without missing values: ^4^P-values=0.6642 without missing: ^5^P-values the same with/without missing values

**eTable 3a:** Mean scores for Anxiety and Depression by diabetes status and tumor site (n=3,512)*

|  |  | **Anxiety** | | | | | **Depression** | | | |
| --- | --- | --- | --- | --- | --- | --- | --- | --- | --- | --- |
| **Tumor Site** | N Res | | No-DM | DM | *P* | N Res | | No-DM | DM | *P* |
| **Oral Cavity and Pharynx** | 100 | | 55.5 (8.46) | 54.6 (7.95) | 0.56518 | 100 | | 50 (6.82) | 50.7 (7.21) | 0.60626 |
| **Digestive System** | 325 | | 54.1 (9.04) | 55.3 (10.15) | 0.27883 | 325 | | 49.9 (8.42) | 50.4 (9.33) | 0.61877 |
| Colon and Rectum | 150 | | 53.2 (9.34) | 54 (8.14) | 0.59413 | 150 | | 48.8 (8.74) | 49.1 (8.26) | 0.82168 |
| Pancreas | 122 | | 55.8 (9.15) | 55.9 (10.82) | 0.97132 | 122 | | 51.7 (7.96) | 50.9 (9.53) | 0.59010 |
| Other^b^ | 53 | | 55.4 (6.81) | 55.5 (10.92) | 0.97011 | 53 | | 52 (6.92) | 51 (10.25) | 0.69719 |
| **Respiratory System** | 216 | | 55.6 (7.81) | 54.6 (7.9) | 0.38887 | 216 | | 52 (7.98) | 51.8 (8.6) | 0.89353 |
| Lung and Bronchus | 193 | | 55.3 (8.07) | 54.8 (7.73) | 0.67114 | 193 | | 52 (8.22) | 52.2 (8.48) | 0.85435 |
| Other^c^ | 23 | | 57.8 (4.65) | 53.2 (9.5) | 0.17866 | 23 | | 52.2 (5.74) | 48.9 (9.49) | 0.34548 |
| **Skin^d^** | 190 | | 51.7 (9.82) | 56 (8.59) | **0.00850** | 190 | | 47.6 (8.48) | 51.4 (8.02) | **0.01085** |
| Melanoma of the skin | 182 | | 51.8 (9.79) | 55.3 (8.09) | **0.02956** | 182 | | 47.6 (8.45) | 50.8 (7.52) | **0.03205** |
| Other^e^ | 8 | | 49.9 (11.74) | 65.1 (11.2) | 0.13507 | 8 | | 46.9 (10.37) | 59.3 (11.51) | 0.20209 |
| **Breast** | 410 | | 54.4 (8.78) | 56.2 (8.37) | 0.08682 | 410 | | 49.6 (8.02) | 53 (8.25) | **0.00078** |
| **Female Genital System** | 191 | | 53.8 (9.84) | 54.7 (8.47) | 0.53847 | 191 | | 49.9 (8.57) | 49.6 (7.82) | 0.84693 |
| Corpus and Uterus | 100 | | 54.9 (10.0) | 55.4 (7.98) | 0.76450 | 100 | | 50 (8.18) | 50.2 (7.54) | 0.92710 |
| Ovary | 68 | | 52.8 (9.08) | 53.5 (9.54) | 0.77098 | 68 | | 49.4 (8.36) | 48.9 (8.01) | 0.81173 |
| Other^f^ | 23 | | 52.3 (11.4) | 54.9 (7.69) | 0.52411 | 23 | | 50.5 (11.16) | 49.4 (9.15) | 0.80159 |
| **Male Genital System** | 447 | | 50.1 (7.52) | 52.8 (7.78) | **0.00325** | 446 | | 46.8 (7.56) | 49.1 (8.02) | **0.01662** |
| Prostate | 425 | | 50 (7.34) | 52.6 (7.76) | **0.00463** | 424 | | 46.7 (7.37) | 49 (7.87) | **0.01514** |
| Other^g^ | 22 | | 53.2 (10.18) | 56.6 (8.03) | 0.46361 | 22 | | 49.3 (10.61) | 50.5 (11.54) | 0.84313 |
| **Urinary System** | 152 | | 53.8 (8.46) | 54.2 (8.01) | 0.75870 | 152 | | 49.1 (8.36) | 50.5 (8.25) | 0.28875 |
| Bladder | 83 | | 53.5 (8.2) | 53.3 (9.0) | 0.92159 | 83 | | 50.3 (8.78) | 49.3 (8.34) | 0.61793 |
| Kidney and Renal Pelvis | 64 | | 54.4 (9.06) | 55.1 (6.73) | 0.72378 | 64 | | 47.4 (8.03) | 51.8 (8.1) | **0.03229** |
| Other^h^ | 5 | | 51.5 (7.23) | 58.7 ( n/a ) | n/a | 5 | | 50.2 (3.79) | 57.3 ( n/a ) | n/a |
| **Brain-nervous system** | 204 | | 55.9 (9.27) | 54.5 (9.33) | 0.33133 | 203 | | 52.3 (8.66) | 51.9 (9.05) | 0.73681 |
| **Endocrine System** | 77 | | 53.3 (9.89) | 53.7 (7.01) | 0.87241 | 77 | | 48.6 (8.33) | 51 (8.23) | 0.27100 |
| Thyroid | 73 | | 53.5 (9.97) | 53.7 (7.2) | 0.93282 | 73 | | 48.8 (8.27) | 51.2 (8.38) | 0.28166 |
| Other^i^ | 4 | | 50.7 (9.45) | 53.7 ( n/a) | n/a | 4 | | 44.9 (10.3) | 46.3 ( n/a ) | n/a |
| **Lymphoma** | 50 | | 53.7 (8.28) | 50.9 (9.97) | 0.32557 | 50 | | 51.4 (8.19) | 47.9 (9.66) | 0.20833 |
| **Myeloma** | 174 | | 51.4 (7.73) | 52.9 (8.31) | 0.21575 | 174 | | 48.2 (8.07) | 50 (7.48) | 0.12504 |
| **Leukemia** | 196 | | 51.2 (8.76) | 51.9 (8.06) | 0.57297 | 196 | | 47.9 (8.69) | 49 (7.91) | 0.41436 |
| **Miscellaneous^j^** | 111 | | 53.6 (9.03) | 56.6 (7.81) | 0.07326 | 111 | | 49.6 (7.89) | 53.4 (9.09) | **0.02775** |

^a^At the end of observation period; Res=Responder; ^b^Esophagus, Stomach, Small Intestine, Liver, Intra Bile Duct, Anus, Gallbladder Other Biliary, Retroperitoneum, Peritoneum, Other Digestive Organs; ^c^Nose, Larynx, Pleura, Trachea, Other; ^d^Excluding Basal and Squamous; ^e^Other Non-Epithelial, Squamous Cell Carcinoma; ^f^Cervix Uteri, Vagina, Vulva, Other Genital Organs; ^g^Testis, Penis, Other Genital Organs; ^h^Ureter, Other Urinary Organs; ^i^Other Endocrine System Organs; ^j^Kaposi Sarcoma, Mesothelioma, Eye Orbit, Soft tissue, Bone Joints, Other Miscellaneous

**eTable 3b:** Mean scores for Fatigue, Pain interference and Physical Function by diabetes status and tumor site (n=3,512)^a^

| Tumor Site | Fatigue | | | | Pain interference | | | | Physical Function | | | |
| --- | --- | --- | --- | --- | --- | --- | --- | --- | --- | --- | --- | --- |
|  | N Res | No-DM | DM | *P* | N Res | No-DM | DM | *P* | N Res | No-DM | DM | *P* |
| Oral Cavity and Pharynx | 100 | 54.3 (9.93) | 56.9 (7.28) | 0.1345 | 100 | 54.9 (9.24) | 57.6 (8.01) | 0.1253 | 101 | 47.4(9.13) | 41.9 (7.19) | **0.001** |
| Digestive System | 325 | 53.6 (9.74) | 56.2 (10.41) | **0.0235** | 325 | 53.9 (9.94) | 54.9 (10.32) | 0.3431 | 326 | 45.7(9.52) | 42.5(9.34) | **0.002** |
| Colon and Rectum | 150 | 51.6(9.33) | 53.6(10.94) | 0.3177 | 150 | 51.8(9.56) | 52.6(10.87) | 0.6822 | 150 | 47.9(8.95) | 43.7(9.35) | **0.0155** |
| Pancreas | 122 | 58.3 (9.77) | 57.6 (10.02) | 0.734 | 122 | 58.3(9.97) | 56.1 (9.91) | 0.249 | 122 | 40.3(9.59) | 41.6(9.11) | 0.4987 |
| Other^b^ | 53 | 54.6 (8.79) | 56.1(10.32) | 0.5825 | 53 | 55.3(9.04) | 55 (10.39) | 0.9083 | 54 | 45.6(8.21) | 43.2(9.95) | 0.3467 |
| Respiratory System | 214 | 56.6 (8.1) | 56.6 (8.93) | 0.9921 | 214 | 55.4 (8.54) | 56.1 (8.72) | 0.5512 | 214 | 40.5(8.96) | 39.3(7.64) | 0.3018 |
| Lung and Bronchus | 191 | 56.6 (8.25) | 57.2 (8.02) | 0.6363 | 191 | 55.1 (8.88) | 56.1 (8.39) | 0.4267 | 191 | 39.9(8.81) | 38.9(7.67) | 0.3881 |
| Other^c^ | 23 | 56.8 (7.83) | 52.3(14.29) | 0.3853 | 23 | 57.9(4.37) | 56.2(11.64) | 0.6667 | 23 | 45.1(9.21) | 42.6(6.87) | 0.4736 |
| Skin^d^ | 191 | 49.2 (9.91) | 56.2 (9.99) | **0.0002** | 191 | 49.8 (9.5) | 56.2 (9.62) | **0.0004** | 191 | 51.3(0.12) | 41.9 (9.07) | **<.0001** |
| Melanoma of the skin | 183 | 49.4 (9.88) | 55.8(10.24) | **0.0012** | 183 | 49.8 (9.5) | 55.8 (9.64) | **0.0015** | 183 | 51.2(10.12) | 42.4(9.25) | **<.0001** |
| Other^e^ | 8 | 44.7 (11.01) | 60.6 (5.44) | **0.0348** | 8 | 49.1 (10.4) | 61.8 (9.02) | 0.1315 | 8 | 54.3(10.88) | 36 (2.67) | **0.0172** |
| Breast | 412 | 52.8 (9.88) | 57.2 (8.99) | **0.0001** | 413 | 51.8 (8.76) | 55.5 (8.68) | **0.0007** | 413 | 48.1(9.6) | 42.9 (8.97) | **<.0001** |
| Female Genital System | 194 | 54.6 (10.26) | 55.3 (8.86) | 0.59993 | 195 | 53.4 (10.07) | 55.4 (8.52) | 0.1473 | 196 | 45 (9.72) | 40.3 (8.14) | **0.0004** |
| Corpus and Uterus | 103 | 53 (10.28) | 55.1 (9.92) | 0.31491 | 104 | 52.8 (10.03) | 54.8 (9.38) | 0.3237 | 104 | 46.2 (9.84) | 39.9(8.68) | **0.001** |
| Ovary | 68 | 56.7 (9.49) | 56.2 (6.79) | 0.80081 | 68 | 53.5 (10.28) | 56.2 (7.15) | 0.1963 | 69 | 43 (9.74) | 39.8 (7) | 0.1163 |
| Other^f^ | 23 | 55.1 (11.78) | 53.4 (9.92) | 0.7094 | 23 | 55.7 (10.04) | 55.3 (9.01) | 0.9215 | 23 | 45.3 (8.75) | 43.4(9.01) | 0.6222 |
| Male Genital System | 446 | 47.8 (9.6) | 53.7 (8.72) | **<.0001** | 447 | 49.1 (8.45) | 52.6 (8.64) | **0.0005** | 449 | 52 (9.15) | 45.4 (7.69) | **<.0001** |
| Prostate | 426 | 47.5 (9.39 | 53.5 (8.79) | **<.0001** | 427 | 49 (8.38) | 52.4 (8.57) | **0.0011** | 429 | 52.2 (9.14) | 45.5 (7.79) | **<.0001** |
| Other^g^ | 20 | 54.4 (1.93) | 56.8 (7.33) | 0.6056 | 20 | 51 (9.88) | 56.9 (9.83) | 0.2876 | 20 | 48.1 (8.66) | 42.6 (5.34) | 0.1215 |
| Urinary System | 152 | 52.1 (9.68) | 56.8 (8.44) | **0.0016** | 152 | 52.8 (9.18) | 56.4 (8.75) | 0.0142 | 152 | 46.9 (9.93) | 41.8 (6.93) | **0.0004** |
| Bladder | 83 | 52.9 (8.24) | 54.8 (8.72) | 0.3032 | 83 | 52.9 (8.88) | 55.6 (9.27) | 0.1871 | 83 | 46.8 (9.87) | 42.5 (6.79) | **0.0223** |
| Kidney and Renal Pelvis | 64 | 50.6 (11.52) | 59.1 (7.63) | **0.0009** | 64 | 51.8 (9.33) | 57.1 (8.19) | 0.0177 | 64 | 48.2 (9.01) | 41 (7.23) | **0.0007** |
| Other^h^ | 5 | 55.9 (7.73) | 63.3 ( n/a ) | n/a | 5 | 59.4 (10.86) | 63.7 ( n/a ) | n/a | 5 | 36.6(14.67) | 41.3 ( n/a ) | n/a |
| Brain-nervous system | 204 | 55.6 (9.54) | 57.7 (8.57) | 0.118 | 204 | 52.9 (9.19) | 53.4 (8.89) | 0.726 | 204 | 45.1(10.64) | 41(10.27) | **0.0086** |
| Endocrine System | 77 | 52 (10.36) | 54.1 (9.57) | 0.4154 | 77 | 47.5 (9.29) | 53 (6.31) | **0.0051** | 79 | 51.8 (8.97) | 48.4(9.29) | 0.163 |
| Thyroid | 73 | 52.2 (10.4) | 53.3 (9.09) | 0.6666 | 73 | 47.6 (9.21) | 52.8 (6.36) | **0.011** | 75 | 51.7 (9.03) | 49.1(8.86) | 0.2823 |
| Other^i^ | 4 | 48.8(11.09) | 69.3 ( n/a ) | n/a | 4 | 45.4 (12.89) | 58.3 ( n/a ) | n/a | 4 | 52.6 (9.6) | 33.7 ( n/a ) | n/a |
| Lymphoma | 51 | 56.3 (7.99) | 54.4 (8.86) | 0.4385 | 51 | 53.5 (8.66) | 51.7 (11.34) | 0.5701 | 51 | 44.7(8.28) | 43.7(12.18) | 0.7354 |
| Myeloma | 174 | 54.5 (8.94) | 56.5 (7.72) | 0.133 | 174 | 54.9 (8.18) | 58.3 (7.28) | **0.0043** | 174 | 43.7(8.2) | 40.3(8.04) | **0.0081** |
| Leukemia | 198 | 52.6 (9.82) | 55.1 (8.84) | 0.0625 | 199 | 51.3 (9.35) | 53.1 (8.51) | 0.1819 | 199 | 48.6(9.11) | 42.7 (8.7) | **<.0001** |
| Miscellaneous^j^ | 112 | 54.2 (9.38) | 58.8 (8.64) | **0.0106** | 113 | 54 (8) | 58 (8.14) | **0.0131** | 114 | 45 (9.58) | 38.2 (7.95) | **<.0001** |

^a^At the end of observation period; Res=Responder; b Esophagus, Stomach, Small Intestine, Liver, Intra Bile Duct, Anus, Gallbladder Other Biliary, Retroperitoneum, Peritoneum, Other Digestive Organs; c Nose, Larynx, Pleura, Trachea, Other; d excluding Basal and Squamous; e Other Non-Epithelial, Squamous Cell Carcinoma; f Cervix Uteri, Vagina, Vulva, Other Genital Organs; g Testis, Penis, Other Genital Organs; h Ureter, Other Urinary Organs; i Other Endocrine System Organs; j Kaposi Sarcoma, Mesothelioma, Eye Orbit, Soft tissue, Bone Joints, Other Miscellaneous

**eTable 4:** Association between Patients Demographics and PRO scores (unadjusted)

|  | **Anxiety** | | **Depression** | | **Fatigue** | | **Pain interference** | | **Physical Function** | |
| --- | --- | --- | --- | --- | --- | --- | --- | --- | --- | --- |
| **Patients Demographics** | β ± SE^#^ | *P* | β ± SE^#^ | *P* | β ± SE^#^ | *P* | β ± SE^#^ | *P* | β ± SE^#^ | *P* |
| **Age** (at CADX) | -0.067 ± 0.01 | **<.0001** | -0.061 ± 0.01 | **<.0001** | -0.017 ± 0.01 | 0.1999 | 0.012 ± 0.01 | 0.3431 | -0.117 ± 0.01 | **<.0001** |
| **Sex** (female) | 2.959 ± 0.33 | **<.0001** | 2.139 ± 0.31 | **<.0001** | 2.86 ± 0.37 | **<.0001** | 1.29 ± 0.35 | **0.0002** | -2.638 ± 0.37 | **<.0001** |
| **Diabetes (yes)** | 1.268 ± 0.35 | **<.0001** | 1.575 ± 0.33 | **<.0001** | 3.778 ± 0.38 | **<.0001** | 3.252 ± 0.36 | **<.0001** | -5.725 ± 0.38 | **<.0001** |
| **Race/Ethnicity** |  |  |  |  |  |  |  |  |  |  |
| Non-Hispanic White | ref |  | ref |  | ref |  | ref |  | ref |  |
| Hispanic | 1.813 ± 0.88 | **0.0396** | 1.238 ± 0.84 | 0.1389 | -1.423 ± 0.98 | 0.1461 | 1.935 ± 0.92 | **0.0362** | -0.449 ± 0.99 | 0.6498 |
| Non-Hispanic non-White | -0.13 ± 0.75 | 0.8627 | -1.129 ± 0.71 | 0.1123 | -0.994 ± 0.84 | 0.2350 | 0.624 ± 0.79 | 0.4280 | 0.527 ± 0.85 | 0.5328 |
| **BMI** | 0.091 ± 0.03 | **0.0009** | 0.113 ± 0.03 | **<.0001** | 0.206 ± 0.03 | **<.0001** | 0.225 ± 0.03 | **<.0001** | -0.287 ± 0.03 | **<.0001** |
| **CCI** | 0.248 ± 0.1 | **0.0161** | 0.328 ± 0.1 | **0.0008** | 0.659 ± 0.11 | **<.0001** | 0.673 ± 0.11 | **<.0001** | -1.167 ± 0.11 | **<.0001** |
| **Married (no)** | 1.854 ± 0.39 | **<.0001** | 2.001 ± 0.37 | **<.0001** | 2.648 ± 0.43 | **<.0001** | 2.06 ± 0.41 | **<.0001** | -2.832 ± 0.44 | **<.0001** |
| **Smoking (yes)** | 1.975 ± 0.35 | **<.0001** | 1.823 ± 0.33 | **<.0001** | 1.587 ± 0.39 | **<.0001** | 2.219 ± 0.37 | **<.0001** | -2.452 ± 0.39 | **<.0001** |
| **Cancer Stage** |  |  |  |  |  |  |  |  |  |  |
| Stage I | ref |  | ref |  | ref |  | ref |  | ref |  |
| Stage II | -1.22 ± 0.52 | **0.0192** | -1.091 ± 0.49 | **0.0267** | -0.553 ± 0.59 | 0.3455 | 0.444 ± 0.55 | 0.4227 | -0.624 ± 0.58 | 0.2827 |
| Stage III | -1.225 ± 0.53 | **0.0222** | -0.601 ± 0.51 | 0.2347 | -0.655 ± 0.6 | 0.2762 | 0.332 ± 0.57 | 0.5602 | -0.399 ± 0.6 | 0.5034 |
| Stage IV | -0.42 ± 0.53 | 0.4249 | 0.021 ± 0.5 | 0.9663 | 2.608 ± 0.59 | **<.0001** | 2.355 ± 0.56 | **<.0001** | -4.188 ± 0.59 | **<.0001** |
| **Cancer treatment** |  |  |  |  |  |  |  |  |  |  |
| Surgery | -0.576 ± 0.44 | 0.1905 | -1.381 ± 0.42 | **0.0009** | -2.551 ± 0.49 | **<.0001** | -2.538 ± 0.46 | **<.0001** | 3.497 ± 0.49 | **<.0001** |
| Chemotherapy | 1.273 ± 0.33 | **0.0001** | 1.354 ± 0.31 | **<.0001** | 3.502 ± 0.36 | **<.0001** | 2.502 ± 0.35 | **<.0001** | -3.689 ± 0.37 | **<.0001** |
| Radiation | 2.081 ± 0.35 | **<.0001** | 1.85 ± 0.34 | **<.0001** | 1.681 ± 0.39 | **<.0001** | 1.1 ± 0.37 | **0.0032** | -1.198 ± 0.4 | **0.0027** |
| Hormone therapy | -0.165 ± 0.4 | 0.6782 | -0.07 ± 0.38 | 0.8523 | 0.194 ± 0.44 | 0.6622 | 0.108 ± 0.42 | 0.7966 | 0.355 ± 0.45 | 0.4284 |
| Immunotherapy | 0.322 ± 0.53 | 0.5419 | 0.674 ± 0.5 | 0.1790 | 1.947 ± 0.59 | **0.0009** | 2.008 ± 0.55 | **0.0003** | -2.142 ± 0.59 | **0.0003** |
| Glucocorticoids | 1.556 ± 0.46 | **0.0008** | 0.317 ± 0.44 | 0.4727 | 0.788 ± 0.52 | 0.1268 | 1.237 ± 0.49 | **0.0110** | -0.949 ± 0.52 | 0.0691 |

**eTable 5:** Association of Diabetes on Patient Reported Outcomes in Patients with Cancer (unadjusted)

|  | **All patients*** (n=3,512) | | | | **Women**^$^ (n=1,724) | | | | **Men**^$^ (n=1,788) | | | |
| --- | --- | --- | --- | --- | --- | --- | --- | --- | --- | --- | --- | --- |
| **Patients Reported Outcomes** | No-DM | DM |  |  | No-DM | No-DM |  |  | No-DM | DM |  |  |
|  | N Res | N Res | β ± SE^#^ | *P* | N Res | N Res | β ± SE^$^ | *P* | N Res | N Res | β ± SE^&^ | *P* |
| **Anxiety** | 1895 | 948 | 1.268 ± 0.35 | **0.0003** | 927 | 434 | 0.632 ± 0.52 | 0.2204 | 968 | 514 | 2.004 ± 0.46 | **<.0001** |
| **Depression** | 1894 | 947 | 1.575 ± 0.33 | **<.0001** | 927 | 433 | 0.902 ± 0.49 | 0.0666 | 967 | 514 | 2.299 ± 0.44 | **<.0001** |
| **Fatigue** | 1901 | 949 | 3.778 ± 0.38 | **<.0001** | 933 | 433 | 2.55 ± 0.56 | **<.0001** | 968 | 516 | 5.051 ± 0.52 | **<.0001** |
| **Pain interference** | 1906 | 949 | 3.252 ± 0.36 | **<.0001** | 936 | 433 | 2.453 ± 0.54 | **<.0001** | 970 | 516 | 4.046 ± 0.49 | **<.0001** |
| **Physical function** | 1911 | 952 | -5.725 ± 0.38 | **<.0001** | 939 | 435 | -5.038 ± 0.55 | **<.0001** | 972 | 517 | -6.511 ± 0.52 | **<.0001** |
